# Spread of *Aedes japonicus japonicus* (Theobald, 1901) in Austria, 2011–2015, and first records of the subspecies for Hungary, 2012, and the principality of Liechtenstein, 2015

**DOI:** 10.1186/s13071-016-1645-8

**Published:** 2016-06-24

**Authors:** Bernhard Seidel, Norbert Nowotny, Tamás Bakonyi, Franz Allerberger, Francis Schaffner

**Affiliations:** Technical Office of Ecology and Landscape Assessment, Nibelungenstrasse 51, A-3680 Persenbeug, Austria; Department of Theoretical Biology, University of Vienna, Althanstrasse 14, A-1090 Vienna, Austria; Viral Zoonoses, Emerging and Vector-Borne Infections Group, Institute of Virology, University of Veterinary Medicine, Veterinaerplatz 1, A-1210 Vienna, Austria; Department of Basic Medical Sciences, College of Medicine, Mohammed Bin Rashid University of Medicine and Health Sciences, Dubai Healthcare City, P.O. Box 505055, Dubai, United Arab Emirates; Department of Microbiology and Infectious Diseases, Faculty of Veterinary Science, Szent István University, Hungária krt. 23-25, H-1143 Budapest, Hungary; Institute for Medical Microbiology and Hygiene, Austrian Agency for Health and Food Safety (AGES), Waehringerstrasse 25a, A-1096 Vienna, Austria; Avia-GIS, Agro-Veterinary Information and Analysis, Risschotlei 33, B-2980 Zoersel, Belgium; National Centre for Vector Entomology, Institute of Parasitology, University of Zurich, Winterthurerstrasse 266a, CH-8057 Zurich, Switzerland

**Keywords:** *Aedes japonicus*, Asian bush mosquito, Invasive mosquito species, Active spread, First record, Austria, Hungary, Bavaria, Liechtenstein

## Abstract

**Background:**

The Asian bush mosquito, *Aedes* (*Hulecoeteomyia*) *japonicus japonicus* (Theobald, 1901) (Diptera: Culicidae), was first identified in Austria in August 2011 in the federal state of Styria at the border to Slovenia.

**Methods:**

Between 2011 and 2015 the spread of *Ae. j. japonicus* was monitored in southern, eastern and western Austrian provinces as well as in neighbouring countries by checking natural and man-made container habitats for the aquatic stages. The search concentrated around the most recent occurrence of *Ae. j. japonicus* and extended up to several kilometres until the subspecies could not be found anymore.

**Results:**

Between May and July 2012 the distribution area of *Ae. j. japonicus* was found to be extended westwards into Carinthia, and eastwards towards the federal state of Burgenland. In August 2012, the subspecies was found in Hungary, representing the first record of an invasive mosquito species in this country. In 2013 its expansion was confirmed at several sites in Austria. Additionally, between April and July 2015, the subspecies was detected in all districts of the westernmost Austrian state Vorarlberg reaching the alpine Montafon valley at the end of October 2015, at all three examined sites in southern Bavaria bordering Vorarlberg, and in the adjacent Principality of Liechtenstein, for which it also represents the first record of an invasive mosquito species. One remarkable finding of the subspecies was located close to the city of Kufstein in the lower Inn valley of the Tyrol in September 2015, which is an isolated occurrence without spatial connection to any known established population.

**Conclusions:**

Our findings demonstrate the ongoing spread of *Ae. j. japonicus* towards all directions within Austria and beyond. Together with the absence of supposed natural barriers, e.g. high mountain chains, at the borders of the current subspecies’ distribution area in south-eastern Austria, these findings suggest a further spread to the Austrian capital Vienna and the Hungarian tourist region of Lake Balaton within the upcoming few years. The observed intrusions in western Austria represent most probably extensions of the population established and spreading in eastern Switzerland and southern Germany. The putative role of the subspecies in pathogen transmission together with its rapid spread observed argues for the implementation of comprehensive nation-wide surveillance and response preparedness.

## Background

Evidence of invasive mosquito populations in zones of temperate climate has extensively been reported for Europe [1]. Since the Asian bush mosquito *Aedes* (*Hulecoeteomyia*) *japonicus japonicus* (Theobald, 1901) (taxonomic nomenclature according to [2]) was found established in Belgium in 2002 [3] and later in Switzerland and southern Germany in 2008 [4], several further colonised areas have been reported from other parts of Europe to date (summarised in [5]).

**Fig. 1:**
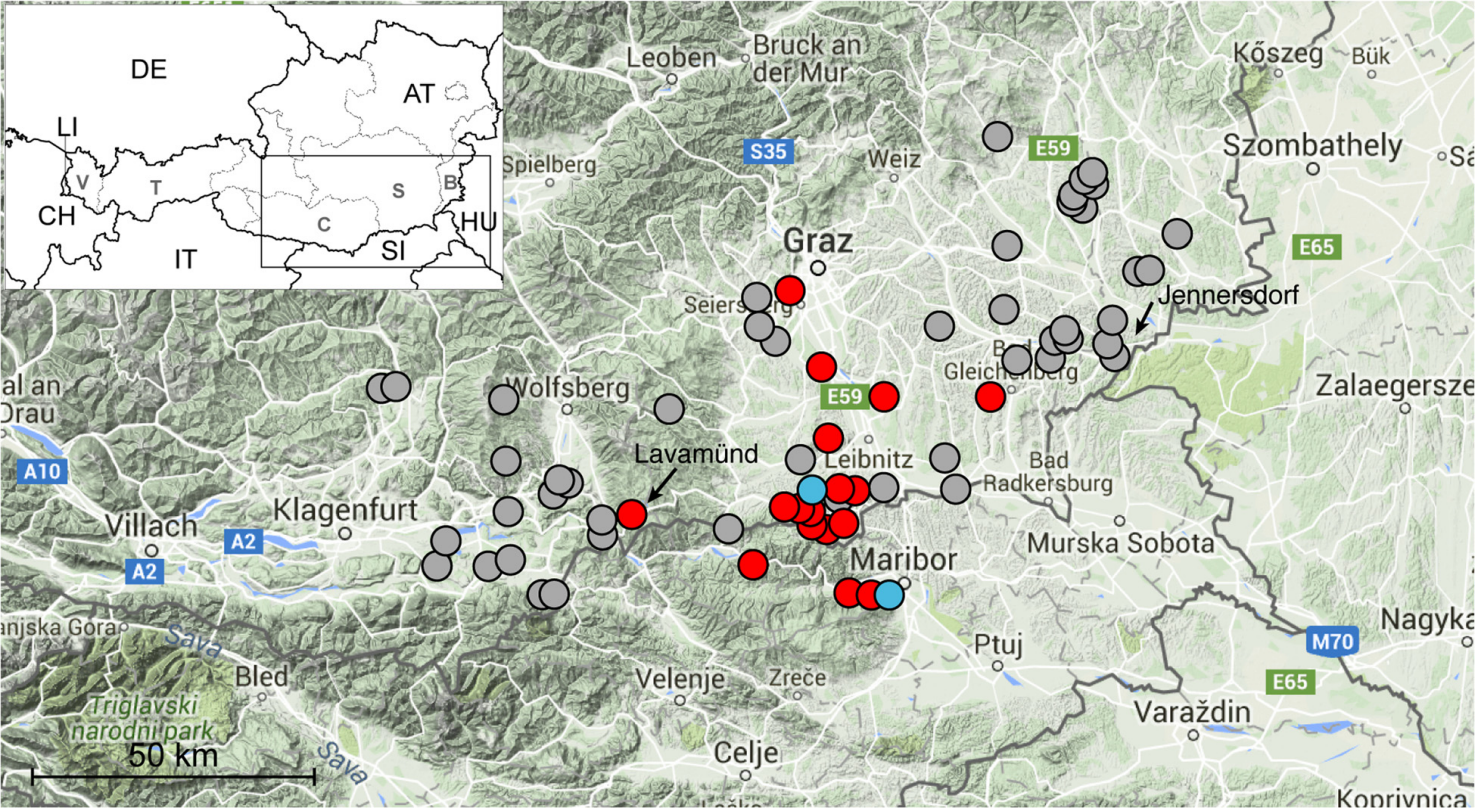
Distribution of *Aedes japonicus japonicus* in southern Austria and Slovenia from 9 August to 25 October 2011. The *black*-and-*white* overview map shows Austria and its neighbouring countries; the detailed map segment is delineated. *Blue circles* indicate the first detection sites of *Ae. j. japonicus* in Slovenia and Austria, respectively; *red circles* mark further detection sites in Austria and Slovenia; *grey circles* indicate sampling sites in which *Ae. j. japonicus* was not detected. The cities of Lavamünd (detection site) and Jennersdorf (absence of the subspecies) are indicated by *black arrows*. *Scale-bar*: 50 km

*Aedes japonicus japonicus* was incidentally discovered in a remote mountainous region in the south of the Austrian province of Styria bordering to Slovenia in August 2011 [6]. Follow-up studies found the subspecies widespread around the city of Maribor in neighbouring Slovenia in September 2011 [6]; however only limited further investigations were carried out there to determine more extensively its distribution [7]. The introduction of *Ae. j. japonicus* into that area could not be backtracked. In October 2011, its distribution in Austria covered an area extending to the Slovenian border southwards and to the Styrian capital Graz northwards. In the neighbouring province of Carinthia, only one specimen of *Ae. j. japonicus* was found near the city of Lavamünd (46.634562N, 14.954196E; 360 m above sea level, m.a.s.l.), located at the border to Slovenia. Despite intensive mosquito sampling around Lavamünd, no further *Ae. j. japonicus* individuals were detected there, and thus this location can be considered the westernmost distribution of the subspecies in Austria in 2011. A similar belt of *Ae. j. japonicus*-negative sites was also observed towards the north and northeast around the 2011 Styrian distribution area (Fig. 1).

Although the subspecies was already reported from several European countries (summarised in [1, 5]), a rapid colonisation of these areas was not generally observed [3, 8]. Thus, in 2011 it was not clear if and how fast further active expansion of the distribution area of *Ae. j. japonicus* would happen and whether new introductions (e.g. through passive transportation) will be necessary to maintain this invasive subspecies in the region [9]. Therefore, we decided to further investigate the area to determine whether *Ae. j. japonicus* is further spreading in south-eastern Austria and could reach the bordering Hungarian and Italian regions. Because of the existence of several separated *Ae. j. japonicus* populations in Switzerland and Germany, we extended our mosquito surveillance activities since autumn 2011 also to the most western Austrian province of Vorarlberg with particular attention to the boundaries of Germany and Switzerland.

## Methods

In August 2011, the *Ae. j. japonicus* surveillance activities were started around the few known breeding sites in southeastern Austria (Styria, Carinthia). In western Austria (Vorarlberg), the surveillance started in 2011 and was intensified in 2015. Inspections were performed at sites that looked suitable for the subspecies, mostly at human settlements and more rarely in forests, by search of immature life stages in container habitats, both man-made (tyres, barrels, vases, etc.) and natural (rock pools, tree holes). Despite artificial water-filled vessels acting as oviposition traps proved to be efficient to survey *Ae. j. japonicus* [10], we choose to use available breeding sites to survey the spread of the species. This allows to observe the ecological behaviour of the species in the local context and avoids repetitive visits (deposit of ovitraps and successive checking) that would have increased the cost of the surveillance. When the survey demonstrated the presence of the subspecies in a given area, we investigated the surroundings up to distant spots where we did not find the subspecies anymore.

Based on the hypothesis of dispersal by traffic transportation, three survey sites were selected in different directions and at distances of approximately 60 km from the sites of first detection in Styria and in Carinthia, respectively. These “control point” sites served as “sentinel” sites in order to detect at an early stage a non-predictable spread by e.g. passive transportation. These three “control points” were located along possible spreading routes like ecological corridors (e.g. valleys of rivers Drau, Glan, and Raab) and major traffic routes, and were examined for immature stages at least three times per season since 2012: (i) Hackerberg/Burgenland as a “control point” towards Vienna (47.195047N, 16.105060E; 299 m.a.s.l.); (ii) Unterloibl in southern Carinthia, as a “control point” towards Italy (46.508390N, 14.290065E; 501 m.a.s.l.); and (iii) Unterbergen-Althofen in northern Carinthia as a “control point” towards western Styria and the River Mur valley (46.876278N, 14.449979E; 606 m.a.s.l.). The bordering Hungarian region was investigated once in 2012.

In western Austria, the surveillance was first focusing on a few places in the federal state Vorarlberg, because of the known presence of a population in neighbouring Switzerland, and its probable spread. Later, following the finding of the subspecies in Vorarlberg’s Hohenweiler, the surveillance was extended into a systematic investigation including the whole of Vorarlberg and also areas in all three neighbouring countries (Bavaria/Germany, Liechtenstein and Switzerland). Besides the designed study, a specific short mission was performed in Liechtenstein by checking putative container habitats in cemeteries; this was planned within the VectorNet project, in order to confirm the presence or absence of *Ae. j. japonicus* in the Principality and thus fill a gap in the VectorNet database and the related species’ distribution map (http://ecdc.europa.eu/en/healthtopics/vectors/vector-maps/Pages/VBORNET_maps.aspx).

Collected immature specimen samples were brought to the laboratory for morphological identification of larvae and emerged adults according to the key by Schaffner et al. [11].

## Results and discussion

### South-eastern Austria and neighbouring regions of Slovenia and Hungary

In **2012** the expansion of the subspecies’ distribution range was investigated in Carinthia **westwards**. Referring to the previous single *Ae. j. japonicus* occurrence detected in Carinthia on 24 September 2011 at the border to Slovenia (Fig. 1), the subspecies’ distribution range was found expanded up to 10 km to the west and northwest by April 2012 (29 April 2012: St. Georgen, 46.714629N, 14.900279E, 499 m.a.s.l.), and up to 27 km by September 2012 (18 September 2012, Stift Griffen, 46.703951N, 14.702619E, 526 m.a.s.l.; 19 September 2012, Eitweg, 46.780202N, 14.893086E, 624 m.a.s.l.).

Towards the **north**, four *Ae. j. japonicus* females were attracted by a human bait created by one of the authors (BS) on 11 July 2012 in the city centre of Graz, the capital of Styria, alongside the main railroad station (Europaplatz, 47.072134N, 15.419154E; 371 m.a.s.l.).

**Fig. 2:**
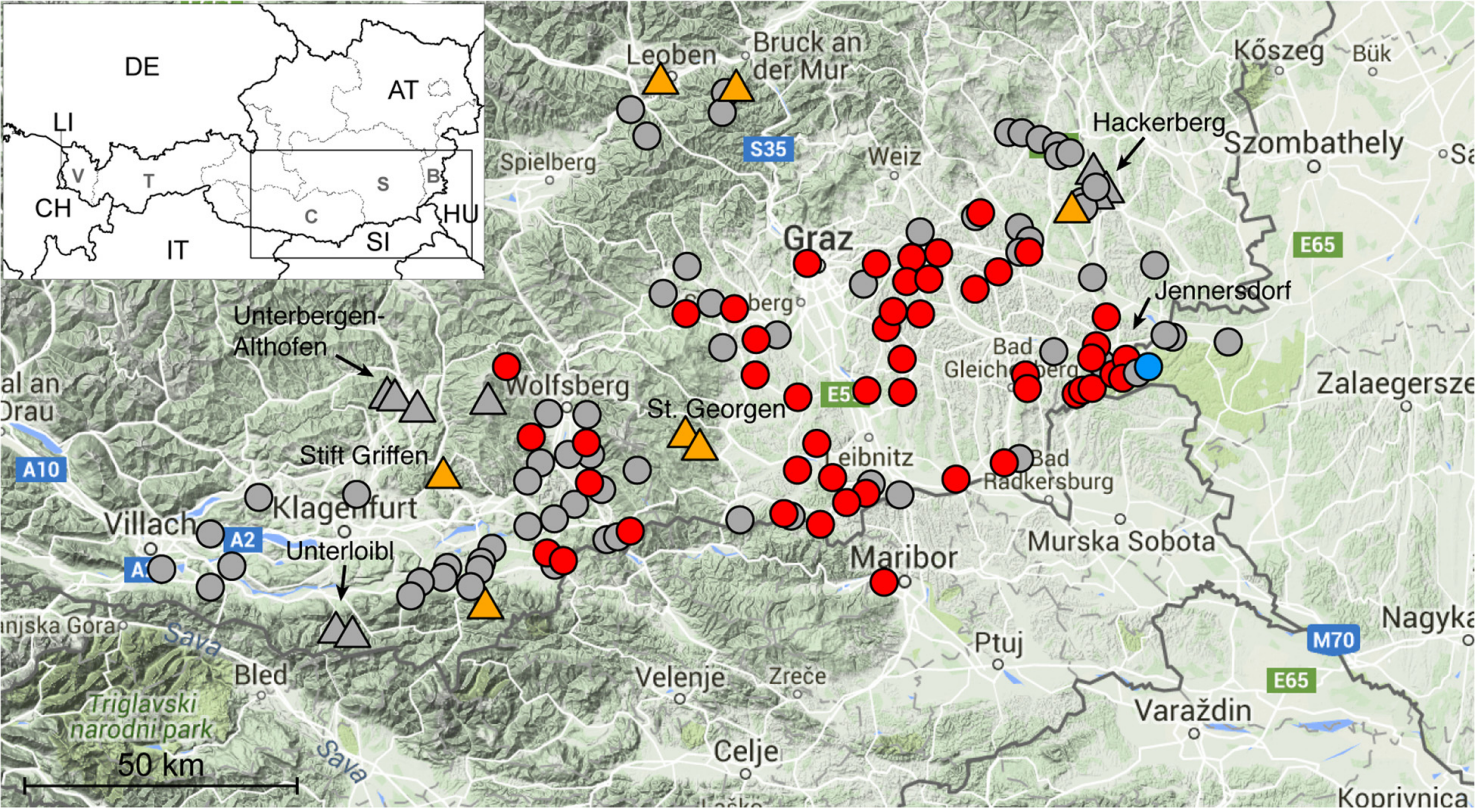
Distribution of *Aedes japonicus japonicus* in southern Austria from 29 April to 9 October 2012, including the first records in Hungary, and from 28 April to 2 November 2013. The *black*-and-*white* overview map shows Austria and its neighbouring countries; the detailed map segment is delineated. The *blue circle* indicates the site of the first detection of *Ae. j. japonicus* in Hungary in 2012; *red circles* mark further detection sites in Austria and Hungary in 2012, *grey circles* indicate sampling sites in which *Ae. j. japonicus* was not detected in 2012. *Orange triangles* indicate detection sites of the subspecies in 2013, *grey triangles* mark sampling sites in which *Ae. j. japonicus* was not detected in 2013. *Black arrows* point towards three “control sites”, i.e. sampling sites established at a certain distance to the first detection sites of the subspecies in Styria and Carinthia, respectively. *Scale-bar*: 50 km

**Eastwards**, several places in the direction towards Hungary were inspected, e.g. around the city of Jennersdorf (46.938036N, 16.1078740E; 245 m.a.s.l.) on 31 August 2011 and again on 29 May 2012, without finding any evidence for the presence of the subspecies. Since 17 July 2012, however, the subspecies was found at several locations around Jennersdorf (Fig. 2). The distance to the Hungarian border from these places is less than 10 km. On 9 August 2012, numerous *Ae. j. japonicus* larvae were found associated with *Culex pipiens* and *Cx. hortensis* in rainwater barrels of two separated sites in Felsöszölnök, Hungary (46.885302N, 16.182786E; 267 m.a.s.l. and 46.874208N, 16.182733E; 314 m.a.s.l.). These represent the first records of an invasive mosquito species and in particular of *Ae. j. japonicus* in Hungary. The occurrence of 50 autochthonous culicid species was recently reported for Hungary [12]. The invasive mosquito subspecies *Ae. j. japonicus* and the Asian tiger mosquito *Aedes* (*Stegomyia*) *albopictus* (Skuse, 1894) were not listed among the Hungarian mosquito fauna, but it was postulated that the latter may appear in Hungary in the future [12]. Interestingly, the species’ border crossing did not occur along a lowland river region that was surveyed, but more southern in a hilly and woody area, which underlines the species’ preferences for bush and woodland landscape.

**Surveillance 2013–2015**: While the “thermal spa tourist” region of eastern Styria and southern Burgenland was already widely colonised by *Ae. j. japonicus* in July 2012, the subspecies was found in the northern parts of this tourist region in early October 2013 (first at our “control point” Hackerberg/Burgenland), midway between its first record in southern Styria and the Austrian capital Vienna. We also confirmed the occurrence of *Ae. j. japonicus* in central Styria around the city of Leoben on 2 November 2013. The western part of Styria might be colonised by the subspecies spreading northward from Carinthia via a pass connection to the city of Neumarkt in Styria (our nearby second “control point” Unterbergen-Althofen in Carinthia proved positive since 28 July 2015). In October 2014 the subspecies was caught for the first time at our third “control point” Unterloibl in southern Carinthia. From there, the subspecies has spread further to Italy, as confirmed by its detection there in 2015 [13].

### Western Austria and neighbouring regions of Germany, Switzerland and Liechtenstein

Since September 2011 mosquito surveillance was also carried out in the westernmost Austrian province Vorarlberg, bordering Germany, Switzerland, and the Principality of Liechtenstein. For example, the area in and around the village of Hohenweiler was checked on 28 September 2011, 27 September 2012, 5 September 2013, 3 October 2013 and 10 October 2014, without evidence for presence of *Ae. j. japonicus*. The same result was obtained in the area of Lustenau, which was for the first time investigated for mosquito larvae in 2014. From 2011 to 2014 only adult *Culex pipiens*, *Cx. hortensis*, and *Ae. geniculatus* were caught in this area. However, on 27 April 2015 twelve 3rd instar larvae specimens of *Ae. j. japonicus* were collected in the village of Hohenweiler, close to the German border, out of two 200 l water vessels, without any other culicid species (except two *Culex* egg-rafts), representing the first detection of *Ae. j. japonicus* in western Austria. From 11 to 13 May 2015 we extended our sampling area to several other districts of Vorarlberg as well as to neighbouring areas of Bavaria (Germany) and Switzerland, and succeeded to detect *Ae. j. japonicus* at numerous locations (Table 1). To add to the VectorNet data base for *Ae. j. japonicus*, sampling was performed in Liechtenstein, by inspecting man-made containers in cemeteries or gardens. On 29 June 2015 the first and only location checked, a cemetery in the village of Schaan, was found to be positive for the subspecies. On 17 July 2015 the subspecies was found in two additional locations in Liechtenstein, i.e. in the villages of Nendeln and Mauren (at a distance of approximately 12 km to Schaan).

**Table 1:** *Aedes japonicus japonicus* findings between 27 April and 17 July 2015 in the westernmost Austrian province of Vorarlberg and adjacent areas of Bavaria (Germany), Switzerland and Liechtenstein

Country/Locality	Date	Latitude	Longitude	Altitude (m.a.s.l.)
Austria				
Hohenweiler^a^	27 April 2015	47.587890N	9.781830E	482
Lustenau	12 May 2015	47.403045N	9.671896E	404
Hörbranz	12 May 2015	47.567728N	9.764382E	416
Lustenau	13 May 2105	47.438850N	9.684357E	395
Bavaria (Germany)				
Scheidegg^a^	11 May 2015	47.591945N	9.845972E	728
Niederstaufen	12 May 2015	47.596926N	9.808601E	576
Niederstaufen	12 May 2015	47.597191N	9.795113E	526
Switzerland				
St. Margarethen	12 May 2015	47.442074N	9.640852E	424
Liechtenstein				
Schaan^a^	29 June 2015	47.168118N	9.513211E	465
Nendeln	17 July 2015	47.200786N	9.545800E	462
Mauren	17 July 2015	47.218708N	9.540176E	460

^a^First records for Vorarlberg, Bavaria and the Principality of Liechtenstein, respectively

*Abbreviation*: *m.a.s.l*. metres above sea level

The last findings of *Ae. j. japonicus* in 2015 were from sampling sites located close to the city of Kufstein (20 September 2015; 47.583840N, 12.154703E; 505 m.a.s.l.) in the lower Inn valley of the Tyrol and up in the alpine Montafon valley of Vorarlberg (10 October 2015; 47.03461N, 009.95174E; 713 m.a.s.l.). Since all known established populations are > 50 km away from the Kufstein location (the closest is the recently identified one in Upper Bavaria/Salzburg [5]), the introduction to this Tyrolean location might have been passive.

**Fig. 3:**
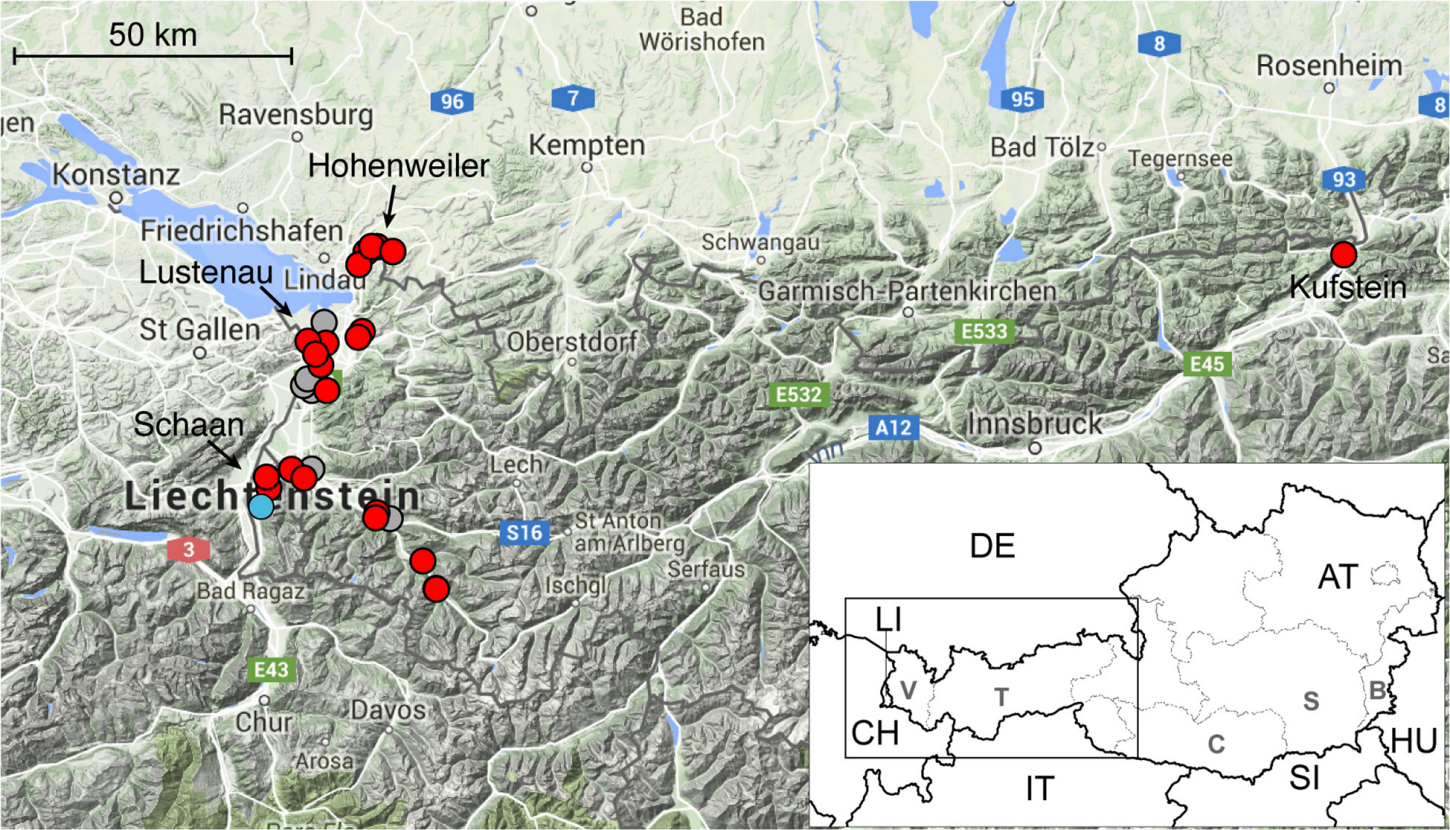
Distribution of *Aedes japonicus japonicus* in western Austria, neighbouring Germany and the Principality of Liechtenstein from 27 April to 20 September 2015. The *black*-and-*white* overview map shows Austria and its neighbouring countries; the detailed map segment is delineated. The *blue circle* indicates the first detection site of *Ae. j. japonicus* in The Principality of Liechtenstein; *red circles* indicate further detection sites in western Austria, Bavaria, and Liechtenstein (*black arrows* point towards important detection sites); *grey circles* mark sampling sites in which *Ae. j. japonicus* was not detected. *Scale-bar*: 50 km

The distribution of *Ae. j. japonicus* in western Austria, neighbouring Germany and the Principality of Liechtenstein in 2015 is shown in Fig. 3.

## Conclusions

Our findings demonstrate an ongoing spread of *Ae. j. japonicus* in Austria towards all directions. The spread into western Austria, observed in 2015, is most likely a result from the active expansion of the population established in Switzerland. The easternmost location of that population was Zurich (Witikon, 47°361944N, 8°603056E) in 2008 [4], and the subspecies was found in other eastern Swiss cantons during the following years (F. Schaffner, unpublished data) up to reaching the Austrian border. Hence it has spread eastwards over around 100 km in 7 years. Also the Principality of Liechtenstein was shown to be colonised in 2015, but first introduction and establishment could have happened there earlier, which can be excluded for the surveyed sites in Vorarlberg, Austria. Meanwhile, *Ae. j. japonicus* has reached northern Italy via the Austrian province of Carinthia [13], Croatia via Slovenia (Merdić, in [14]), and north-eastern France spreading from neighbouring Switzerland [15]. It will probably reach the Austrian capital Vienna and the Hungarian tourist region of Lake Balaton within the upcoming few years.

*Aedes j. japonicus* is not considered a mosquito subspecies posing a major risk in terms of pathogen transmission [1, 16]. Thus, little is done to control its populations in most European countries. However, this mosquito has the potential to play a role in transmission of pathogens like flaviviruses of the Japanese encephalitis complex (West Nile, Japanese encephalitis, and Saint Louis encephalitis viruses) [1, 16]; recent findings support a major role in transmission of La Crosse virus in North America [17]. Moreover, the subspecies can pose a nuisance in forested areas [F. Schaffner, unpublished data], and it was identified as candidate bridge vector for WNV transmission in Switzerland, based on abundance data, spatio-temporal activity, host preferences, and laboratory vector competence for WNV [18]. This, together with the rapid spread observed, argues for the implementation of long term surveillance and response preparedness as well as application of adapted and sustainable control measures in order to limit further spread and to reduce the abundance of this invasive mosquito subspecies [19].
